# Supplementary material for: Effects of high-intensity interval training on strength, speed, and endurance performance among racket sports players: A systematic review
Source: PLoS One. 2024 Jan 5;19(1):e0295362. doi: 10.1371/journal.pone.0295362 (PMC10769056; doi:10.1371/journal.pone.0295362)
Supplement: S1 Data — (DOCX) [file pone.0295362.s002.docx]

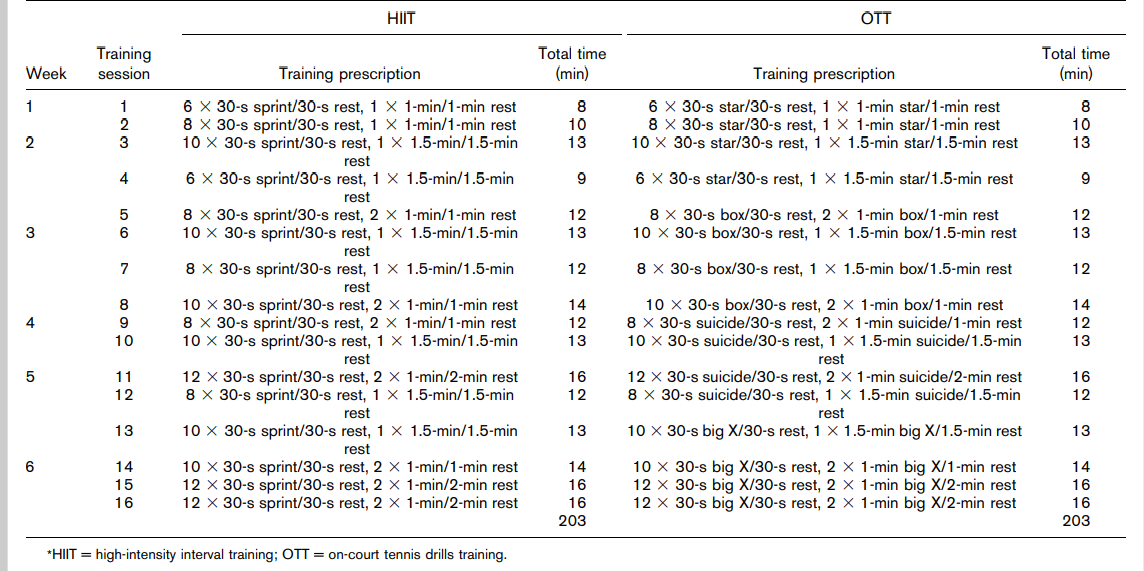


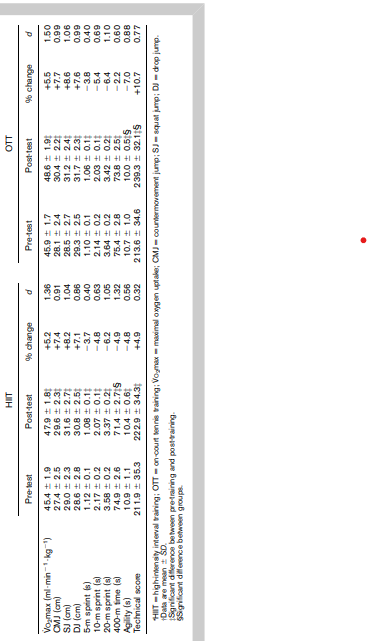


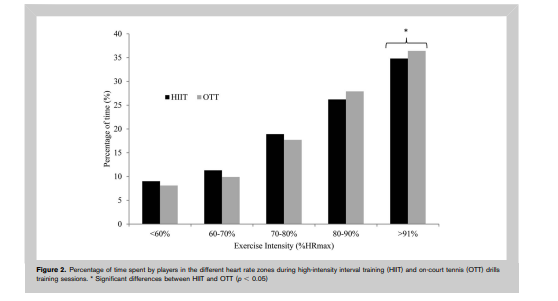
[1]


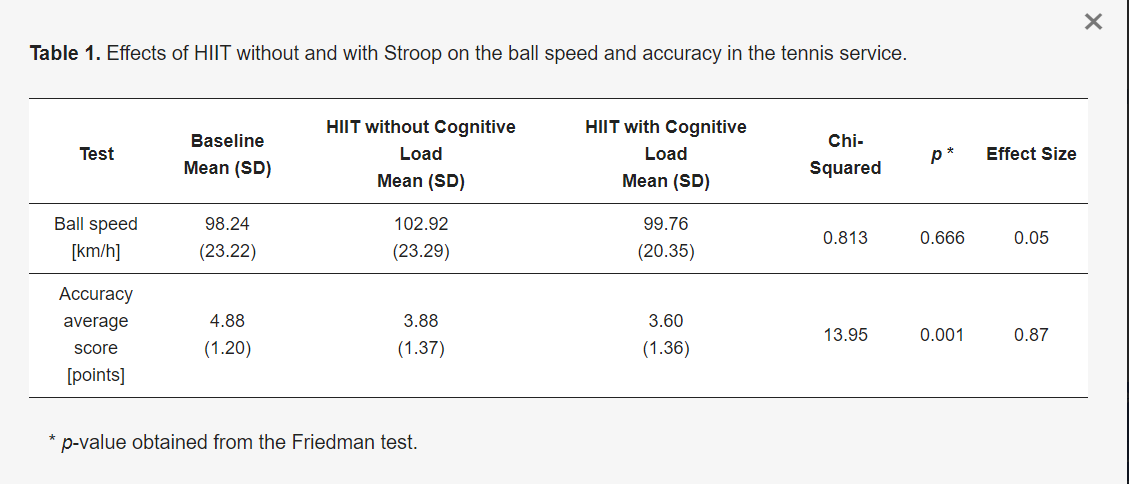


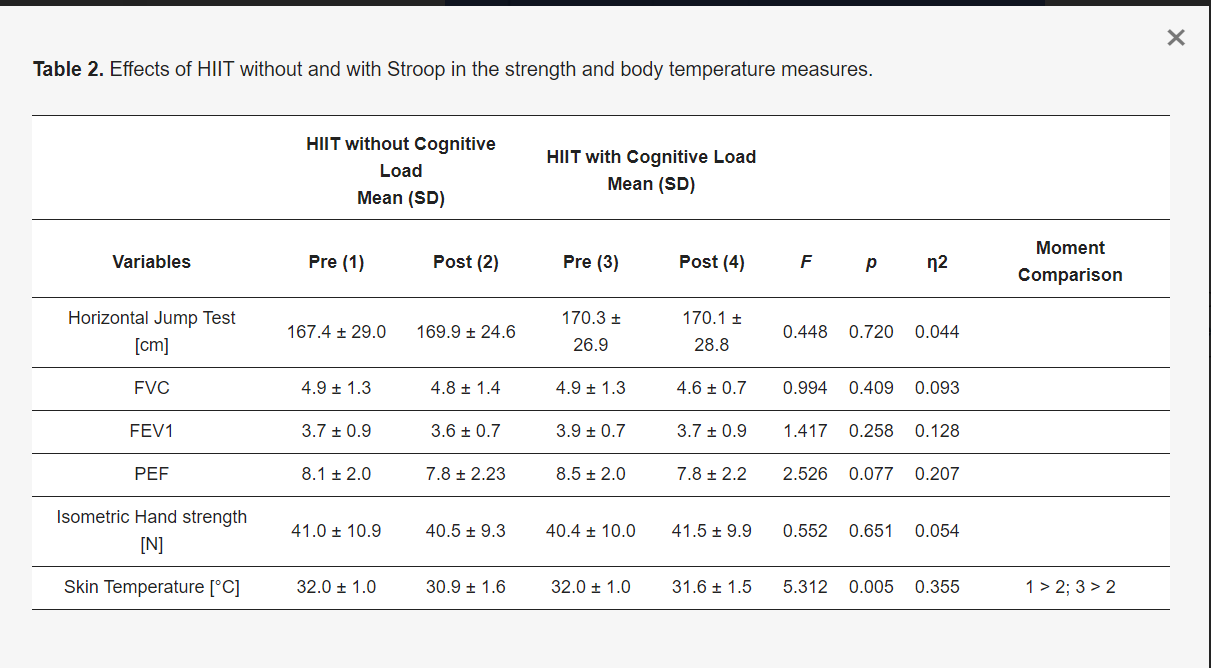


[2]


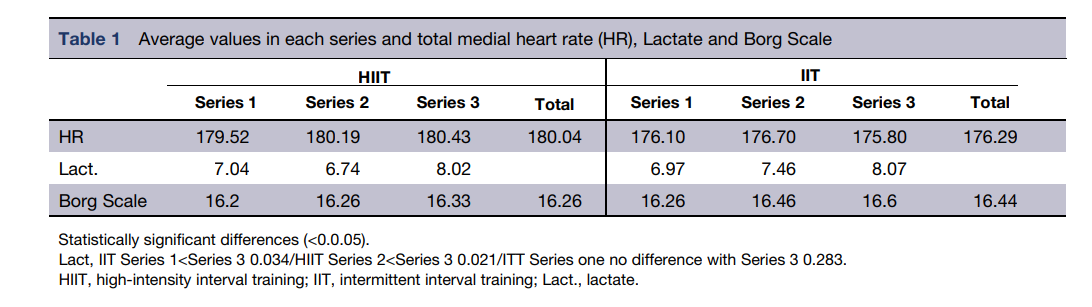


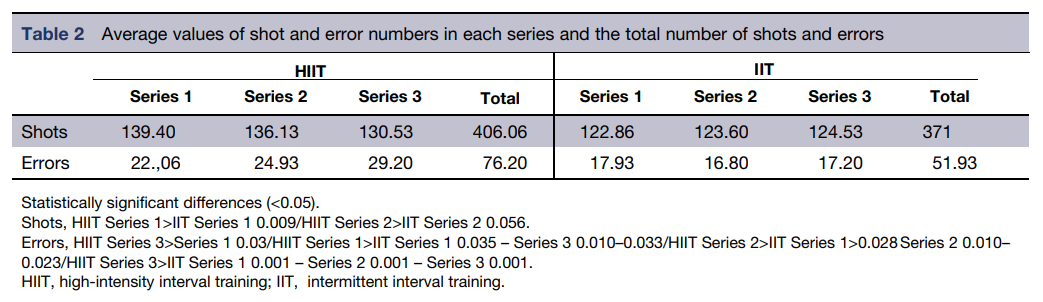


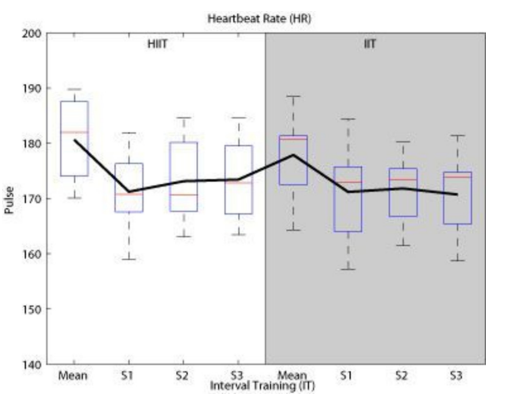


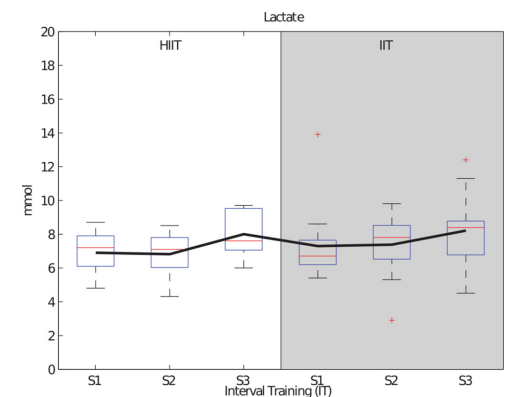


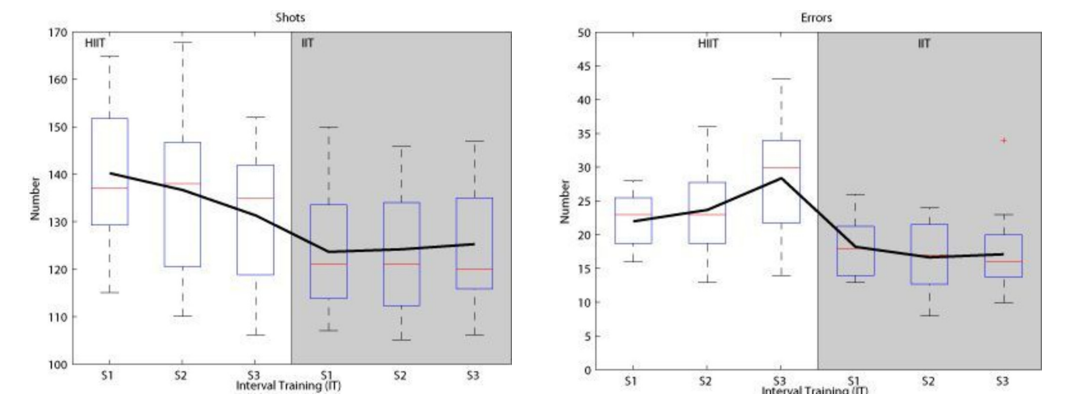


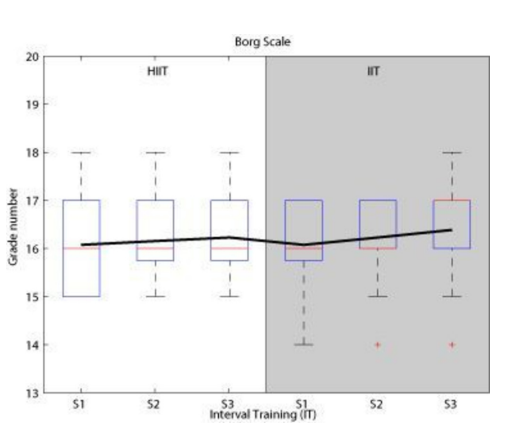


[3]


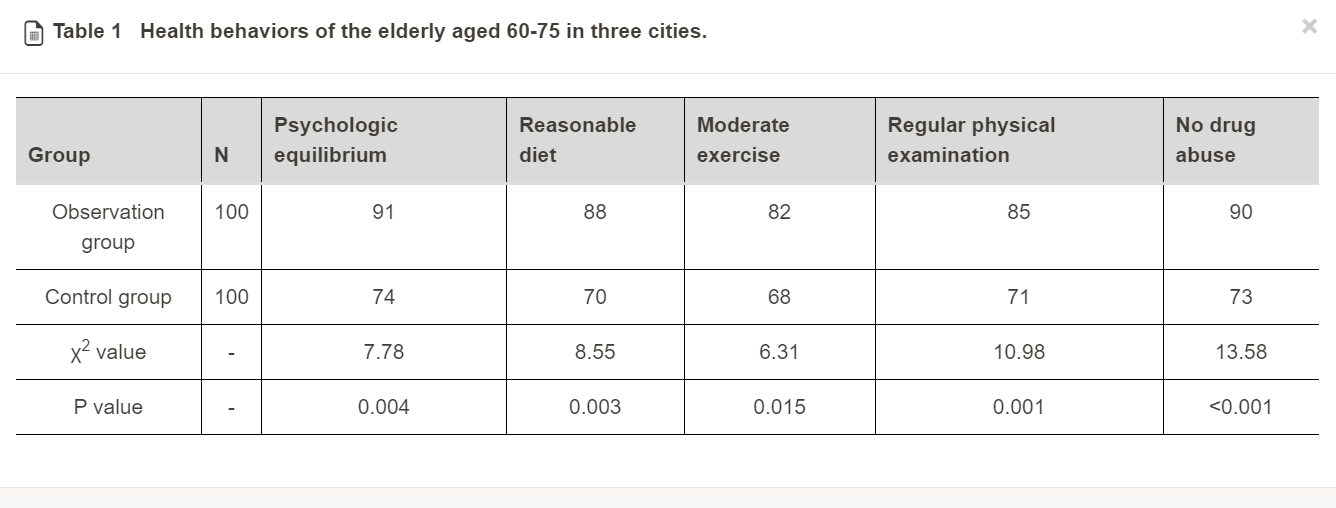


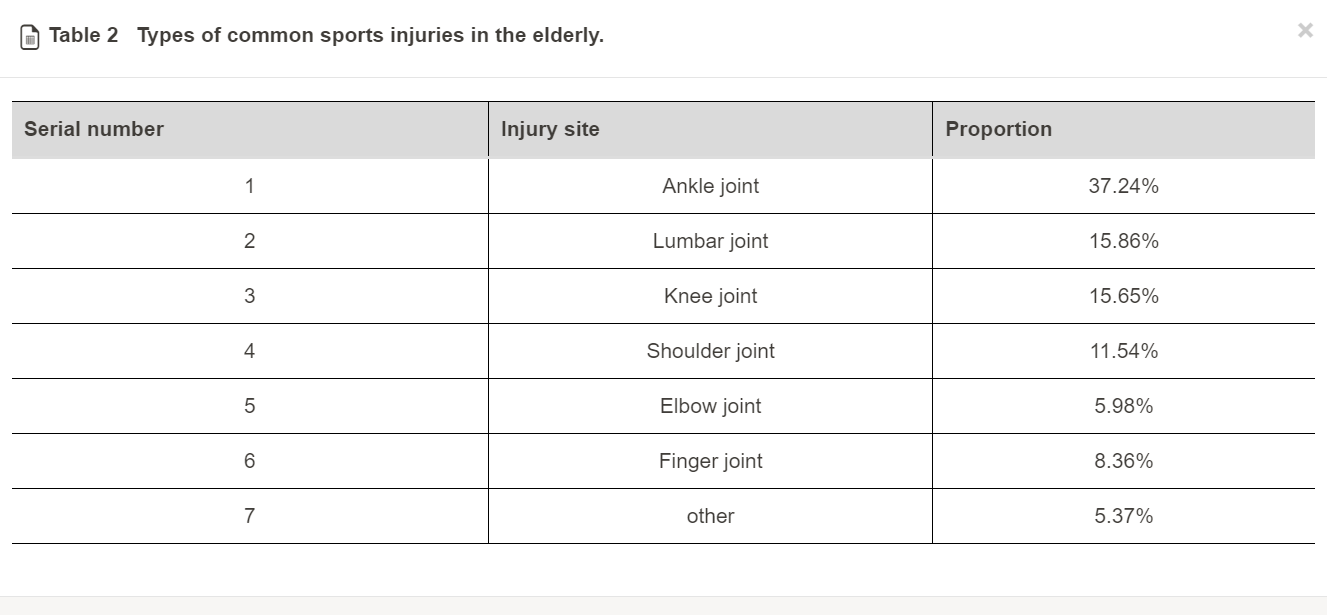


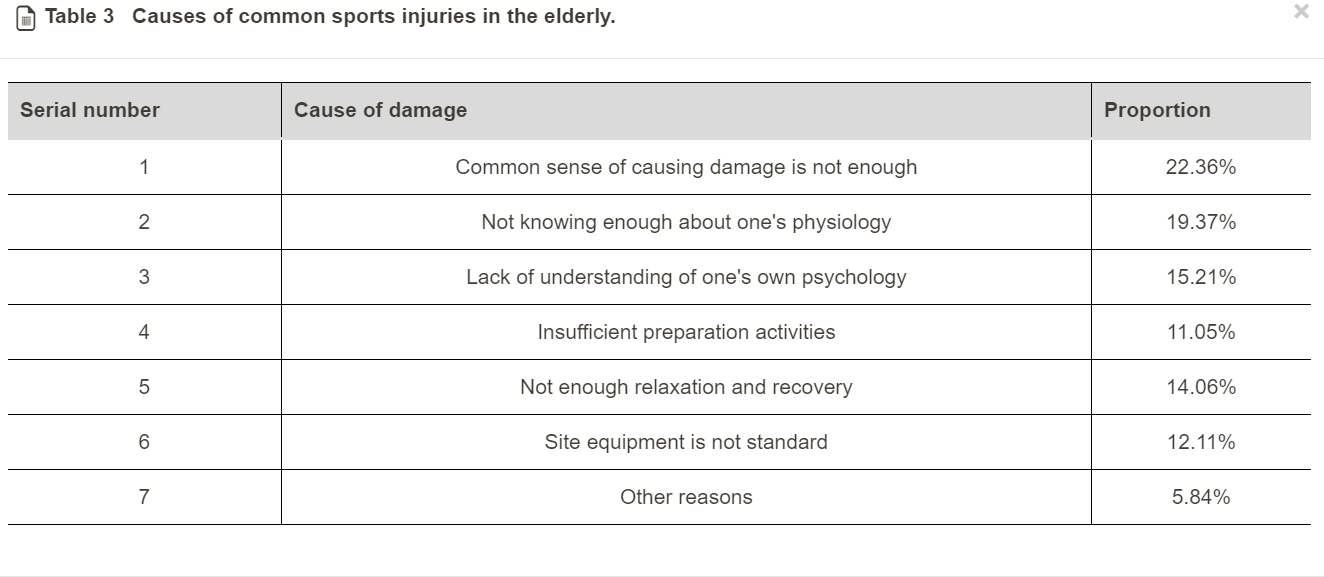


[4]


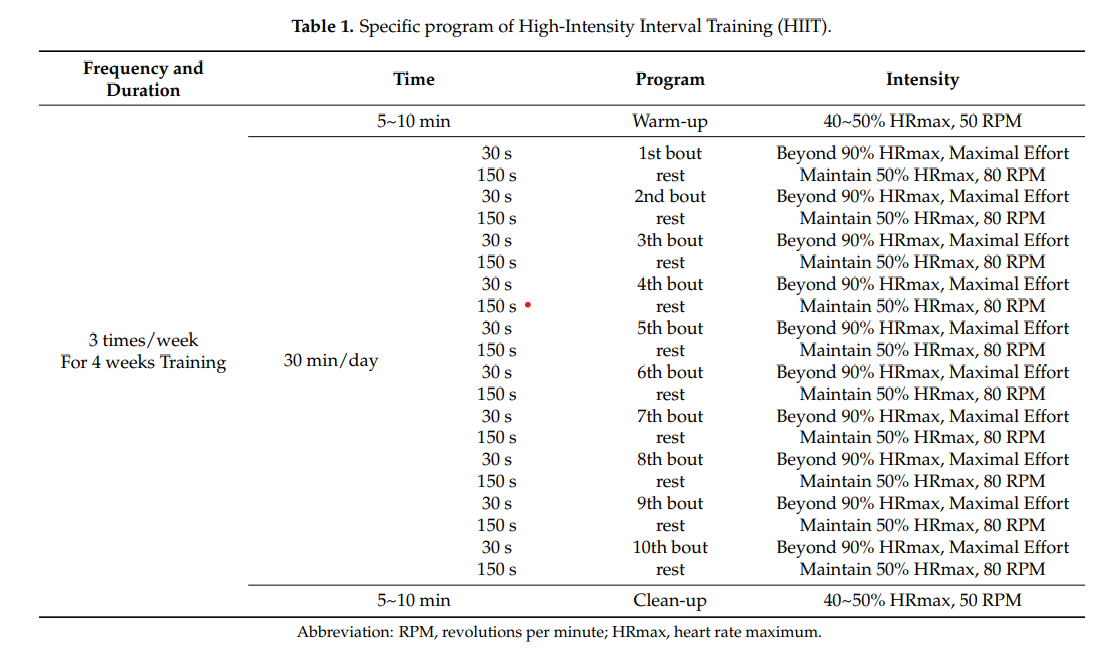


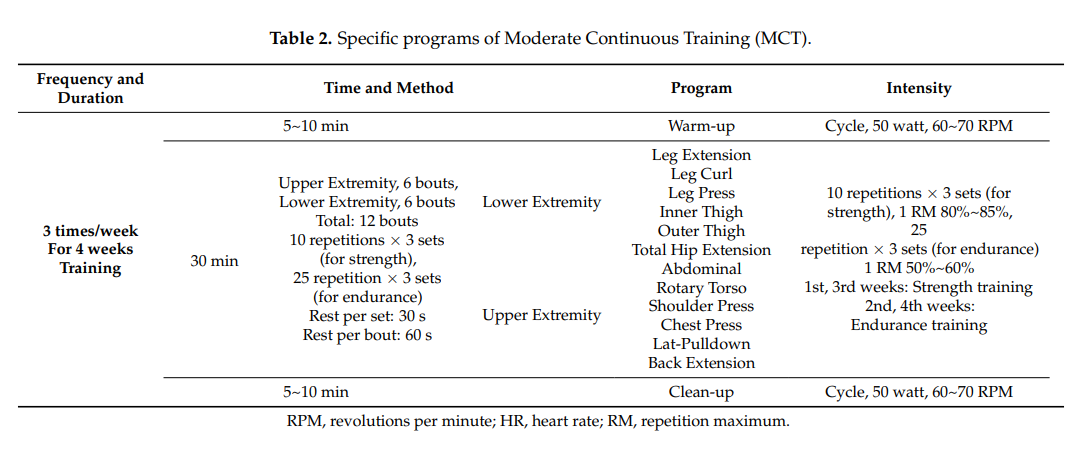


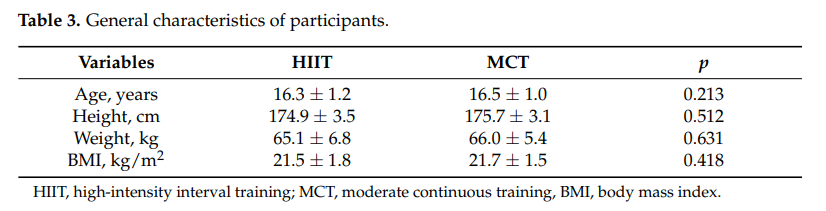


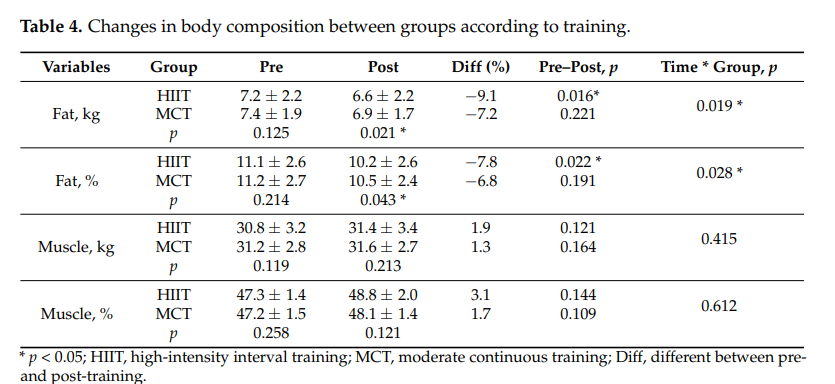


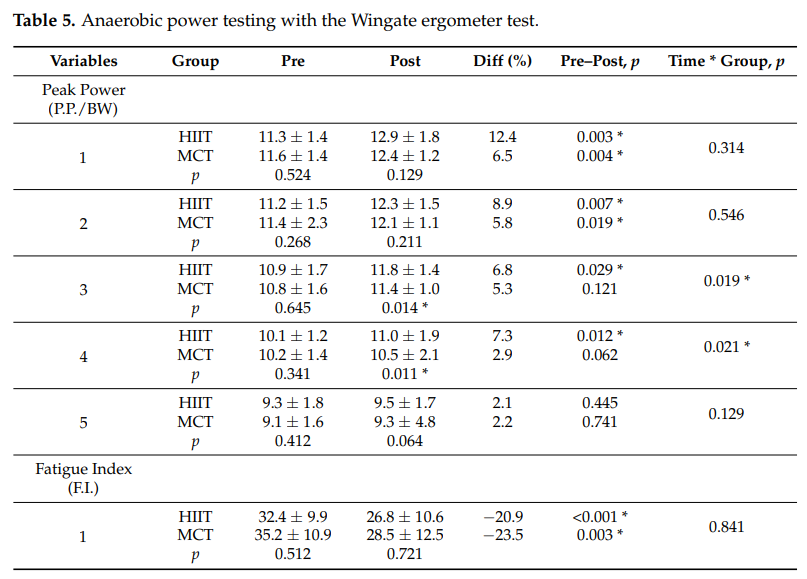


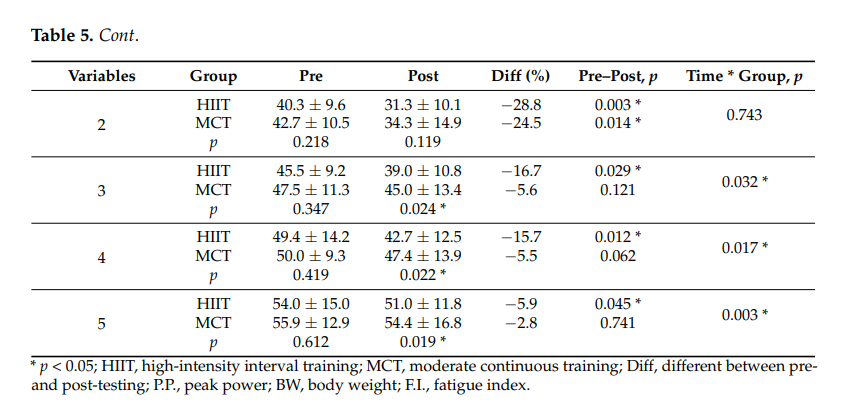


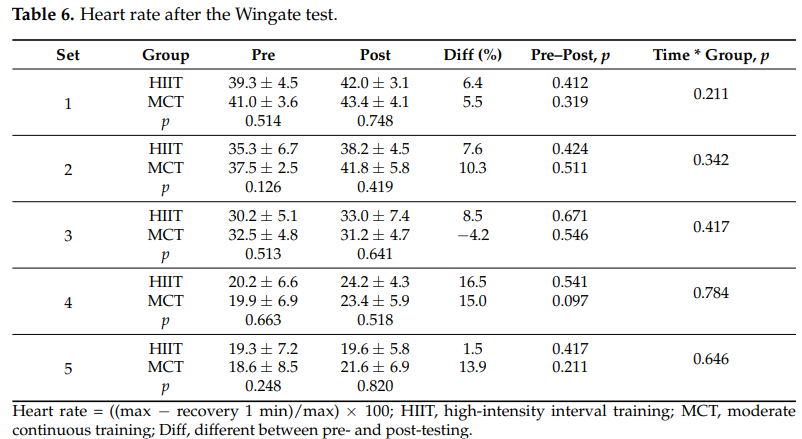


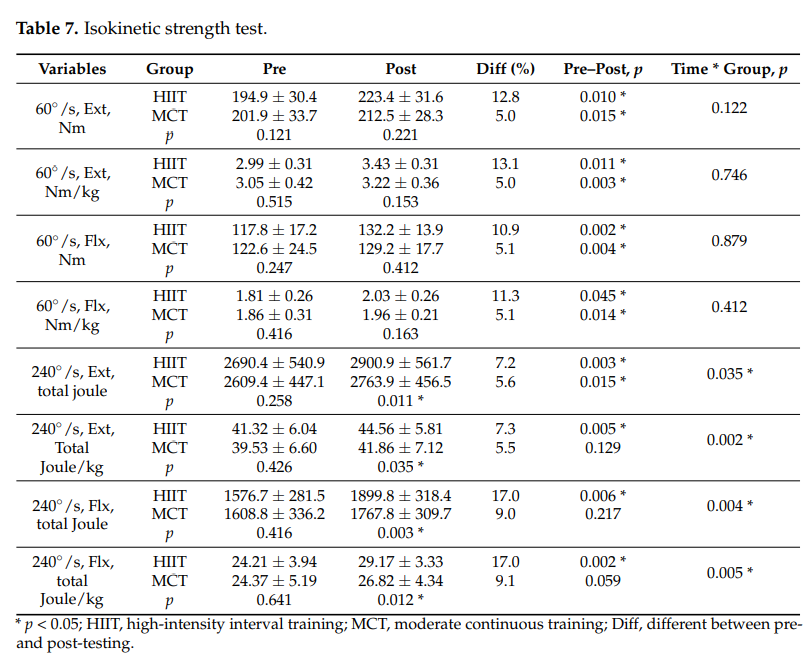


[5]


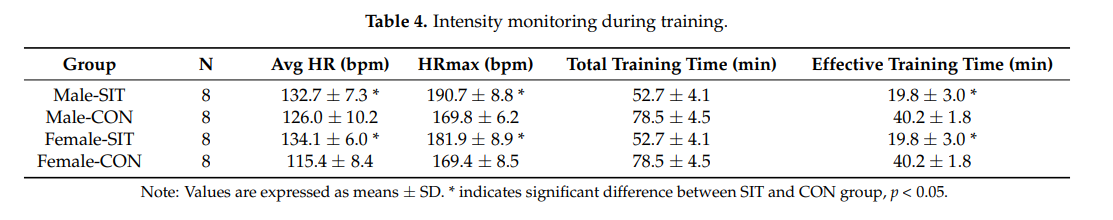


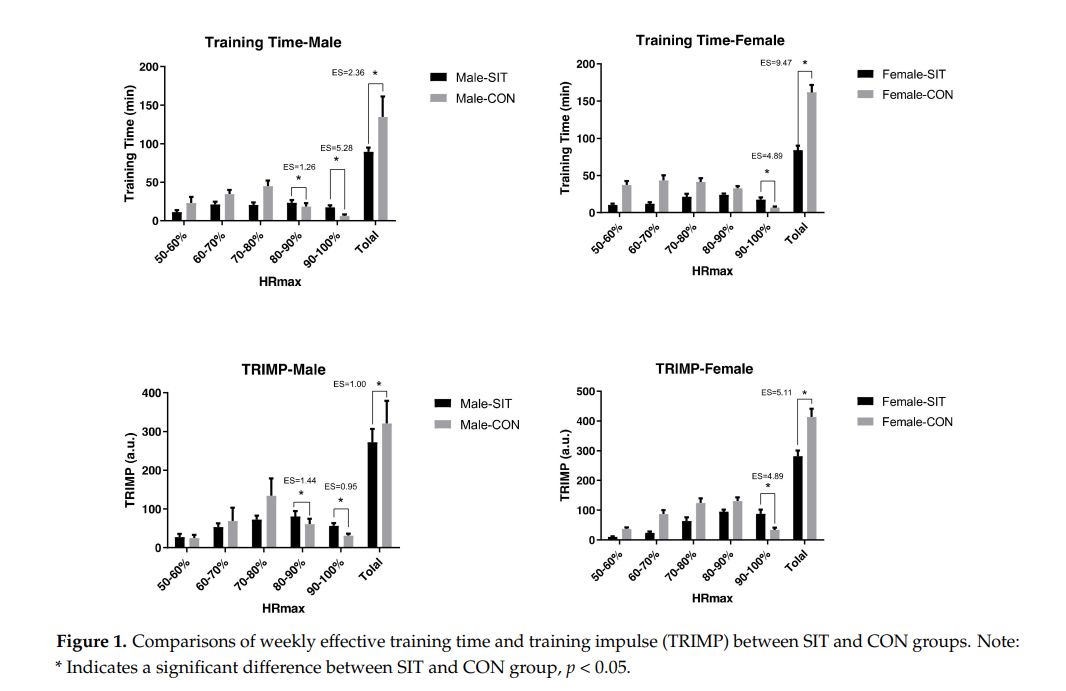


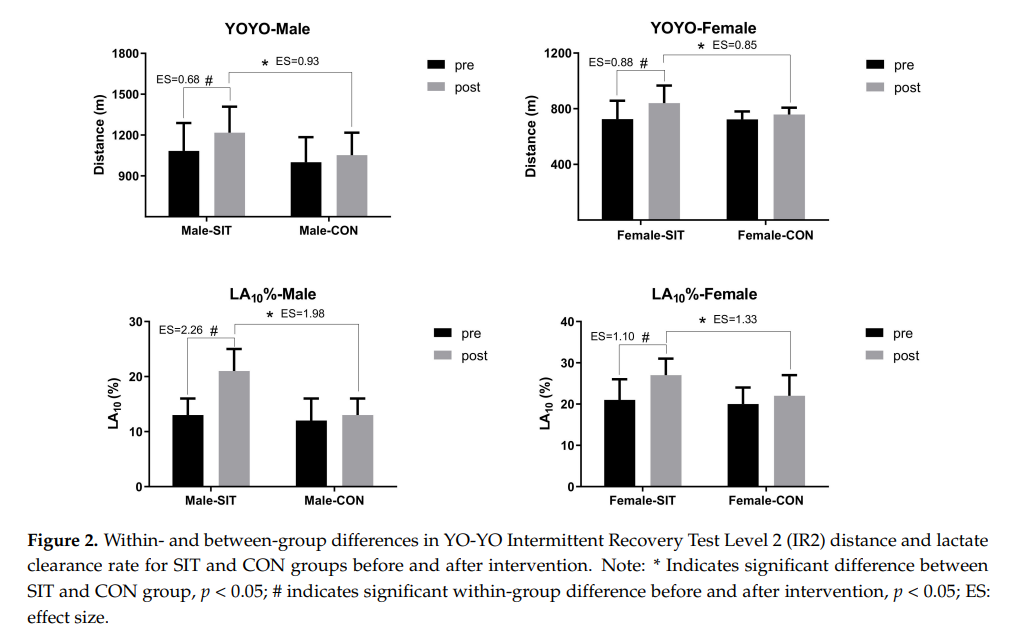


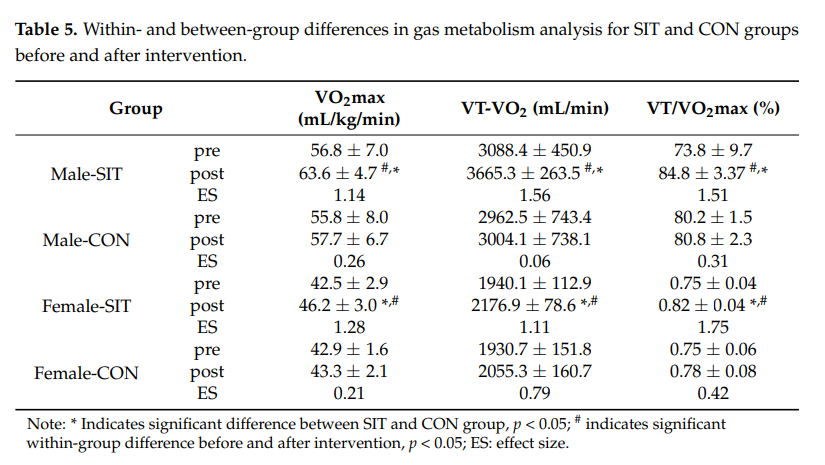


[6]


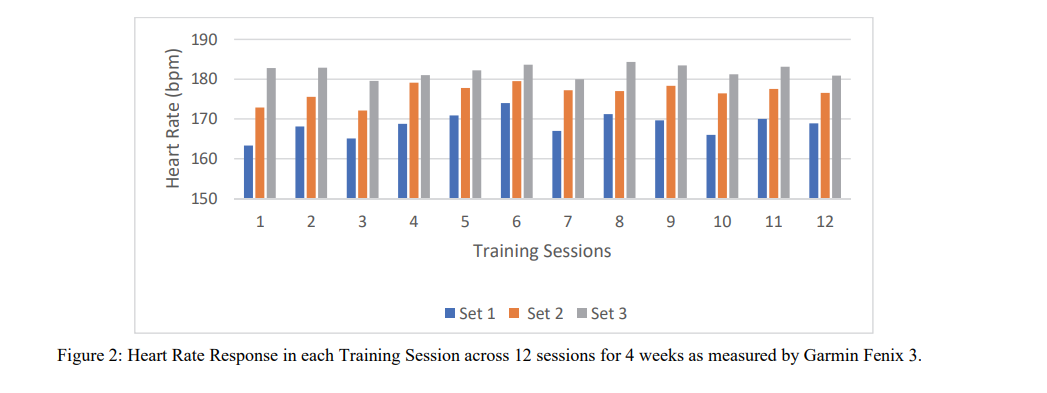


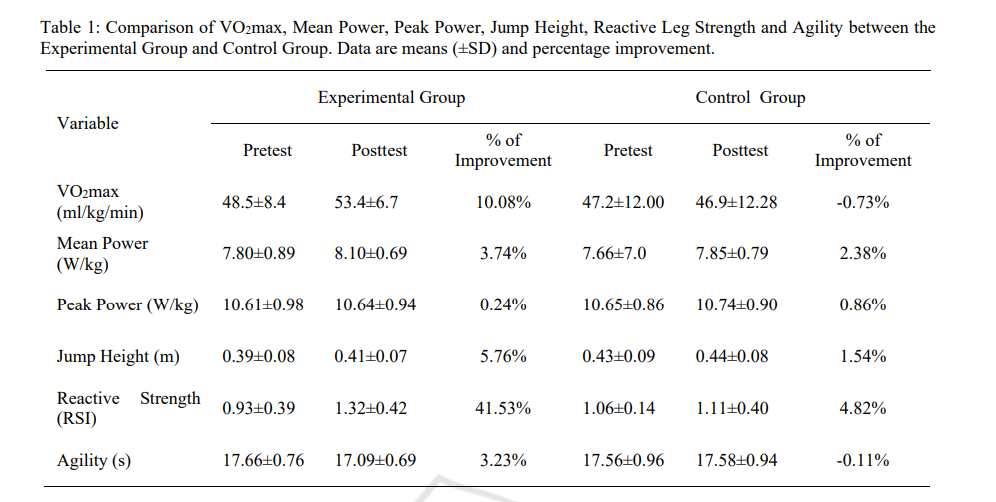


[7]


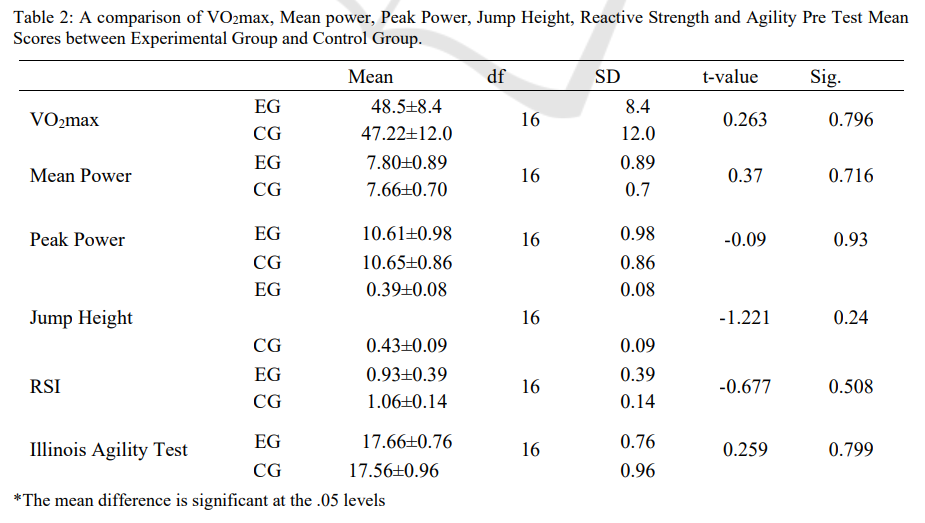


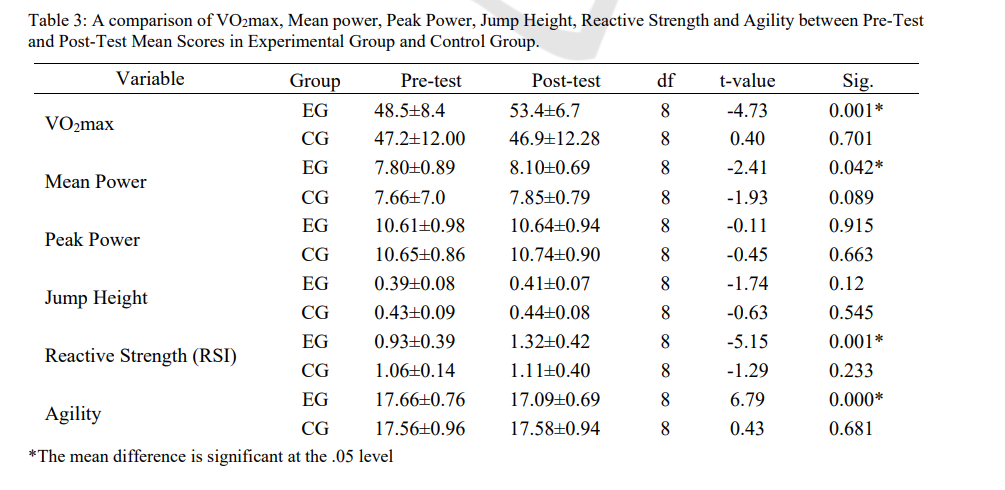


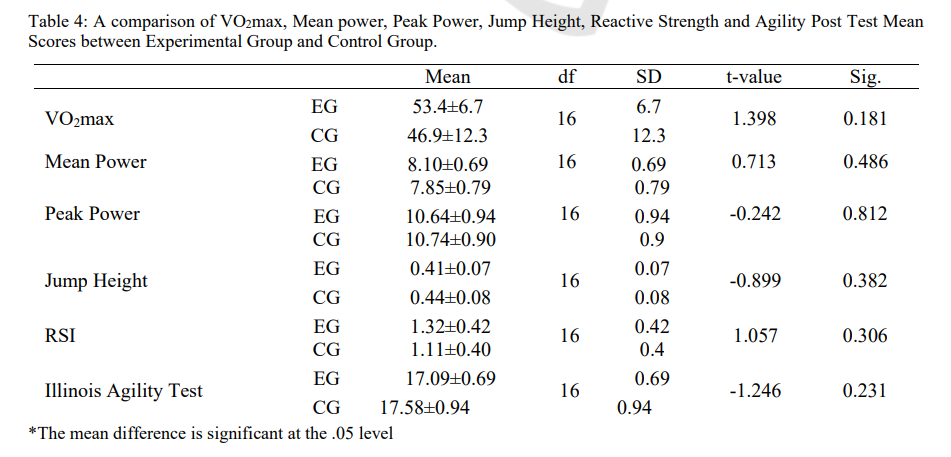


[8]


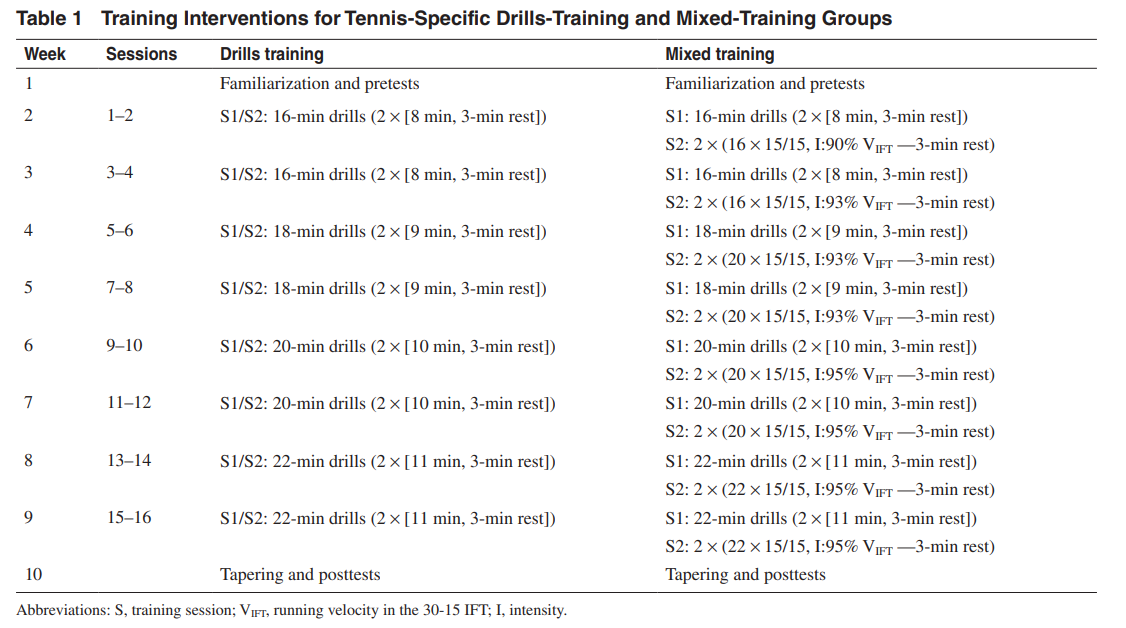


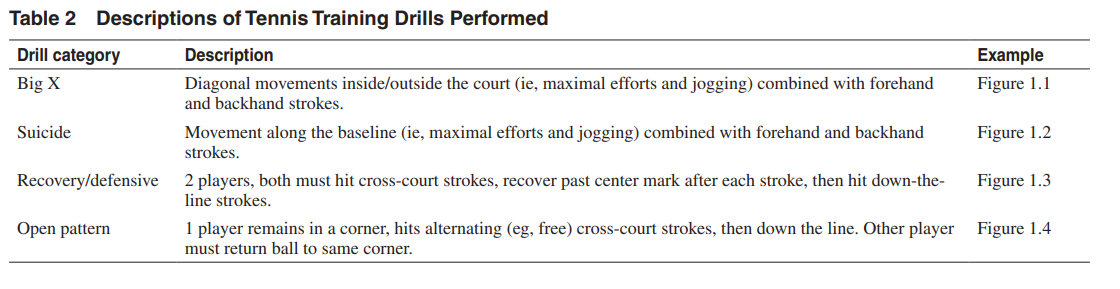


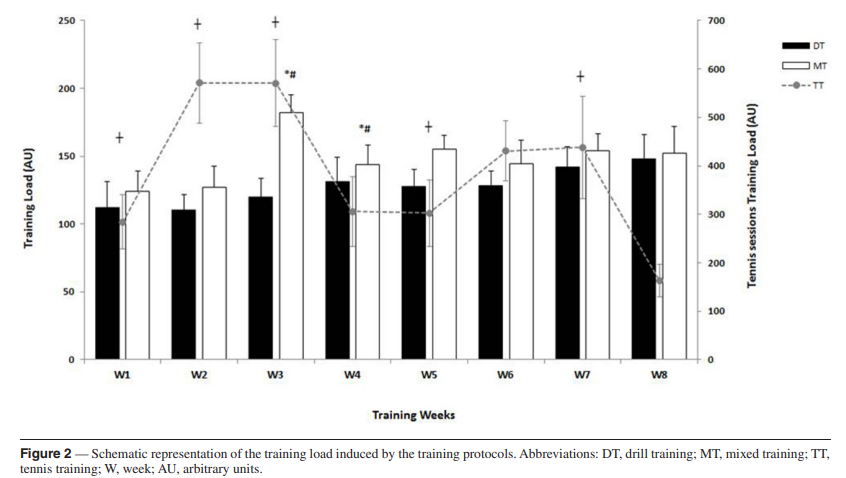


[9]


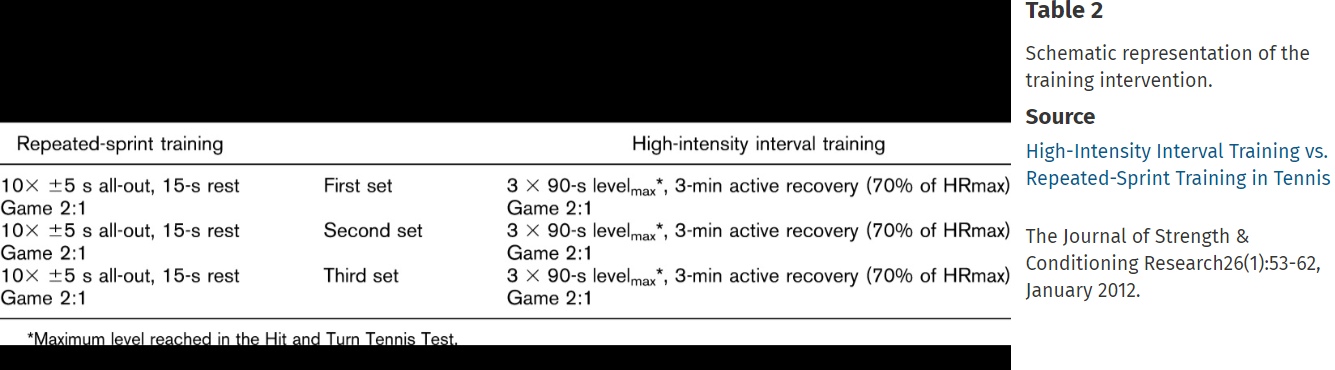


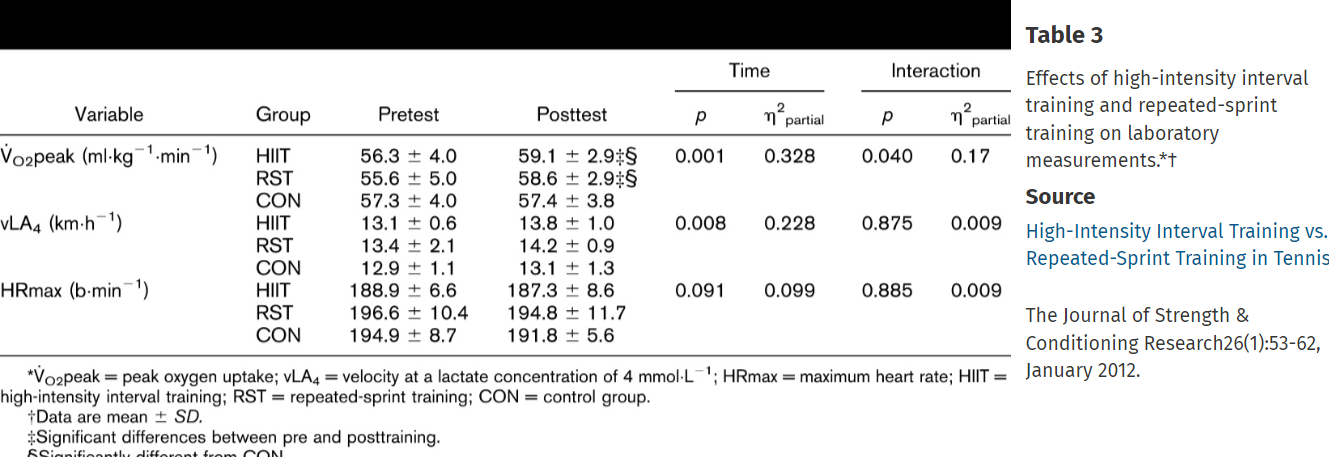


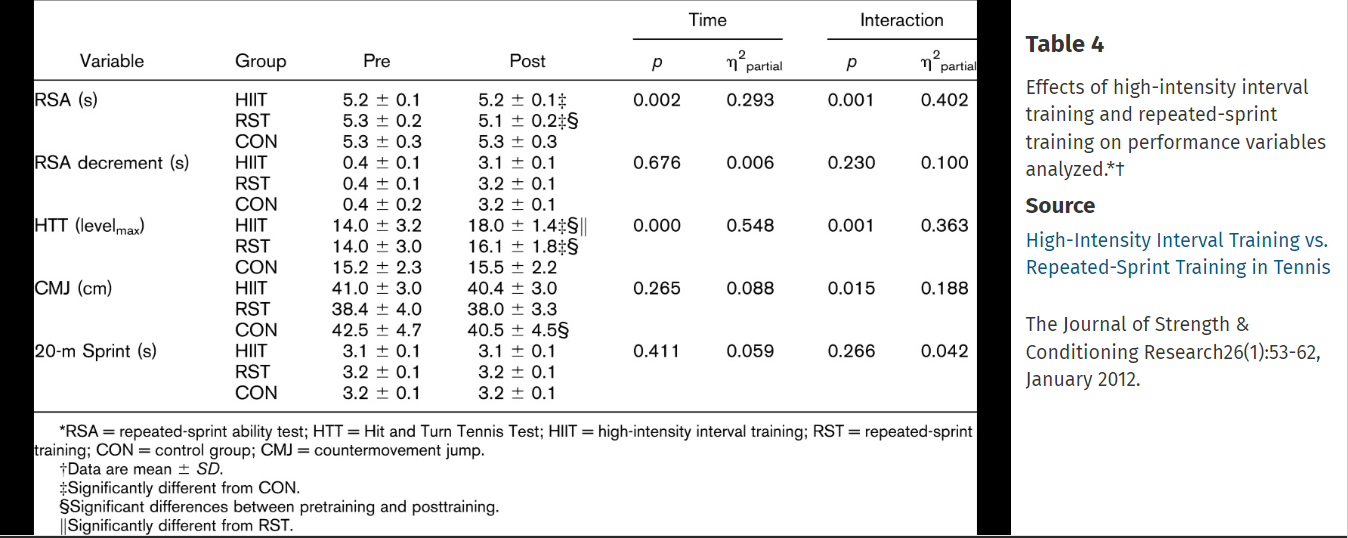


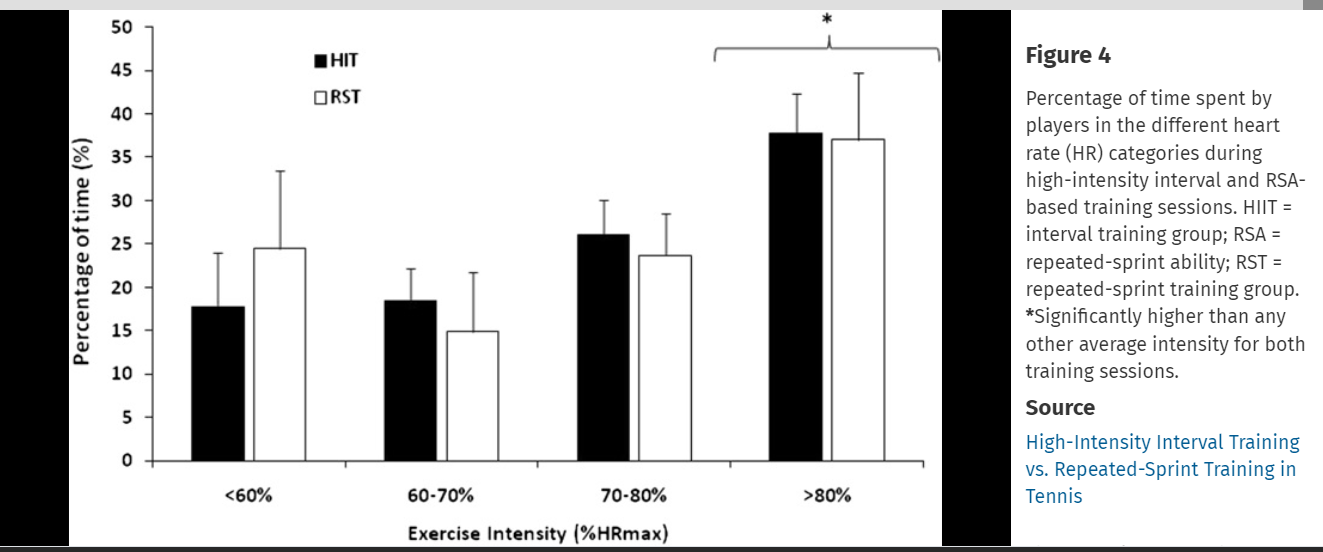


# 【10】

1. Kilit B, Arslan E. Effects of high-intensity interval training vs. on-court tennis training in young tennis players. *The Journal of Strength & Conditioning Research*. 2019 Jan 1;33(1):188-96.
2. Fuentes-García JP, Díaz-García J, López-Gajardo MÁ, Clemente-Suarez VJ. Effects of Combined HIIT and Stroop on Strength Manifestations, Serve Speed and Accuracy in Recreational Tennis Players. *Sustainability.* 2021 Jul 10;13(14):7717.
3. Rodríguez DS, del Valle Soto M. A study of intensity, fatigue and precision in two specific interval trainings in young tennis players: high-intensity interval training versus intermittent interval training. *BMJ Open Sport & Exercise Medicine.* 2017 Aug 1;3(1):e000250.
4. Chen J, Wang J. Influence of high-intensity interval training on table tennis players. *Revista Brasileira de Medicina do Esporte.* 2022 Oct 20;29.
5. Ko DH, Choi YC, Lee DS. The effect of short-term wingate-based high intensity interval training on anaerobic power and isokinetic muscle function in adolescent badminton players. *Children.* 2021 May 31;8(6):458.
6. Liu H, Leng B, Li Q, Liu Y, Bao D, Cui Y. The effect of eight-week sprint interval training on aerobic performance of elite badminton players. *International Journal of Environmental Research and Public Health.* 2021 Jan;18(2):638.
7. Wee EH, Low JY, Chan KQ, Ler HY. Effects of High Intensity Intermittent Badminton Multi-Shuttle Feeding Training on Aerobic and Anaerobic Capacity, Leg Strength Qualities and Agility. *InicSPORTS* 2017 Oct (pp. 39-47).
8. Wee EH, Low JY, Chan KQ, Ler HY. Effects of specific badminton training on aerobic and anaerobic capacity, leg strength qualities and agility among college players. InSport Science Research and Technology Support: 4th and 5th International Congress, icSPORTS 2016, Porto, Portugal, November 7-9, 2016, Revised Selected Papers 4 2019 (pp. 192-203). *Springer International Publishing.*
9. Fernandez-Fernandez J, Sanz D, Sarabia JM, Moya M. The effects of sport-specific drills training or high-intensity interval training in young tennis players. *International journal of sports physiology and performance.* 2017 Jan 1;12(1):90-8.
10. Fernandez-Fernandez J, Zimek R, Wiewelhove T, Ferrauti A. High-intensity interval training vs. repeated-sprint training in tennis. *The Journal of Strength & Conditioning Research.* 2012 Jan 1;26(1):53-62.
