# Supplementary material for: Effects of high-intensity interval training on strength, speed, and endurance performance among racket sports players: A systematic review
Source: PLoS One. 2024 Jan 5;19(1):e0295362. doi: 10.1371/journal.pone.0295362 (PMC10769056; doi:10.1371/journal.pone.0295362)
Supplement: Attachment — Submitted filename: MM_Review PONE D-23-29988.pdf [file pone.0295362.s003.pdf]

Review of the article **"Effects of high-intensity interval; training on strength, speed and endurance performance among racket sports players: a systematic review"**

General Note

In this manuscript, the authors aimed to systematically review the literature on the influence of high-intensity interval training on the strength, speed, and endurance performance of racket sports athletes. After applying the search strategy at four databases and on google scholar and reference lists, the authors included 10 studies in their review. After analyzing the results, the authors emphasizes HIIT as a strategy to improve overall athletic performance comparing to others training methods. The manuscript fits the Plos One scope. Follow bellow some general notes and minor concerns for each section.

General Concerns

Introduction

The introduction session is very extensive. The authors dedicate several pages to approach the problem, justify the investigation and expose the gaps in the specific literature. I agree is utmost important that we have to conduct a "story telling" and "sell the idea". However, research papers must be objective, precise and direct to the point. It is not the case of the introduction session. The authors used 3 paragraphs to explain HIIT effects on racket sports and, surprisingly, in none of these paragraphs the authors explain what is HIIT. My suggestion to the authors is to collapse the 3 paragraphs in one.

At introduction section, the sentence "Racket athletes often rely on the ATP-CP energy system for short energy bursts,…". In fact, to perform a specific movement, it is expected that the phosphagens systems were the prominent system to offer energy, however, during all exercise session, the body will migrate from one system to another. As a suggestion, maybe the authors agree to adjust the sentence and be more specific.

Finally, at the end of the introduction section, the authors state that "…research focusing specifically on racket sports remains limited. Notably, there is a lack of systematic reviews examining the physical effects of HIIT on athletes in racket sports." I believe this sentence is ambiguous since if research in rackets sports remains limited, it is not possible to conduct a robust review with conclusive findings. Maybe the authors consider rewriting the sentences.

Methods

At this section, the authors make a common mistake when trying to review the literature with high-intensity interval exercise. Specific literature on HIIT attested that 11 variables could be manipulated to build a HIIT session. In this sense, is extremely difficult to aggregate HIIT studies to a common analysis since the several possibilities to organize a HIIT protocol. It was expected that at some part of the review, the authors demonstrate and explore the HIIT protocols of the 10 selected. Maybe the author agrees to include in table 3 the adopted protocols. Further, I suggest including why the terminology High-intensity interval training was the chosen one, since it is possible to find studies applying "high intensity interval exercise"; "high intensity intermittent training"; "high intensity intermittent exercise"; "repeated sprint training"; "sprint interval exercise"; among other possibilities.
