# Supplementary material for: Effects of high-intensity interval training on strength, speed, and endurance performance among racket sports players: A systematic review
Source: PLoS One. 2024 Jan 5;19(1):e0295362. doi: 10.1371/journal.pone.0295362 (PMC10769056; doi:10.1371/journal.pone.0295362)
Supplement: Attachment — Submitted filename: Response to Reviewers.docx [file pone.0295362.s004.docx]

Dear Editors,

I hope this letter finds you well. I would like to express my gratitude for the thorough review and valuable feedback provided by you and the reviewers on my manuscript titled "Effects of High-intensity Interval Training on Strength, Speed, and Endurance Performance among [Racket Sports](https://www.topendsports.com/sport/racquet-sports.htm) Players: A Systematic Review." I appreciate the time and effort invested in the evaluation of my work, and I have carefully considered all the comments and suggestions.

I would like to address each of the points raised by the academic reviewers in a systematic manner to clarify and, where appropriate, revise the manuscript in line with their recommendations:

**Response to Reviewer #1**

Dear Professor,

I wish to express my gratitude for your recognition and the thoughtful suggestions you have provided. I would like to offer a concise clarification and an update on the implementation of your suggestions.

*Page 1: use shortened versions throughout the text (High-Intensity Interval Training) (HIIT)*

**The abbreviation has been changed**

*Page 1: Write which racquet sports they are. “table tennis, tennis, and badminton”, add key words or abstract to the section*

**Already added to keywords**

*Page 2: Reference numbers in the all text are written incorrectly. Check and correct reference numbers throughout the text.*

**The number has been rechecked**

*Page 27: use the short form (High-Intensity Interval Training) (HIIT)*

**The abbreviation has been changed**

*References section: check the References (1, 34, 75 control and check the fonts)*

**Reference has been modified**

**Response to Reviewer #2**

Dear Professsor,

I appreciate your thorough review; it is truly an honor. And i have implemented revisions in the manuscript in accordance with your suggestions, and I will provide clarifications below:

*Introduction*

*The introduction session is very extensive. The authors dedicate several pages to approach the problem, justify the investigation and expose the gaps in the specific literature. I agree is utmost important that we have to conduct a “story telling” and “sell the idea”. However, research papers must be objective, precise and direct to the point. It is not the case of the introduction session. The authors used 3 paragraphs to explain HIIT effects on racket sports and, surprisingly, in none of these paragraphs the authors explain what is HIIT. My suggestion to the authors is to collapse the 3 paragraphs in one.*

*At introduction section, the sentence “Racket athletes often rely on the ATP-CP energy system for short energy bursts,…”. In fact, to perform a specific movement, it is expected that the phosphagens systems were the prominent system to offer energy, however, during all exercise session, the body will migrate from one system to another. As a suggestion, maybe the authors agree to adjust the sentence and be more specific.*

*Finally, at the end of the introduction section, the authors state that “…research focusing specifically on racket sports remains limited. Notably, there is a lack of systematic reviews examining the physical effects of HIIT on athletes in racket sports.” I believe this sentence is ambiguous since if research in rackets sports remains limited, it is not possible to conduct a robust review with conclusive findings. Maybe the authors consider rewriting the sentences.*

**I have incorporated an introduction to HIIT within the introductory section and have combined the three relevant paragraphs into a single unified paragraph, as per your guidance. Additionally, I have provided a comprehensive explanation of the connection between racket players and the ATP-CP energy system. Furthermore, I have addressed the sentence modification you highlighted at the end of the text.**

*Methods*

*At this section, the authors make a common mistake when trying to review the literature with high-intensity interval exercise. Specific literature on HIIT attested that 11 variables could be manipulated to build a HIIT session. In this sense, is extremely difficult to aggregate HIIT studies to a common analysis since the several possibilities to organize a HIIT protocol. It was expected that at some part of the review, the authors demonstrate and explore the HIIT protocols of the 10 selected. Maybe the author agrees to include in table 3 the adopted protocols. Further, I suggest including why the terminology High-intensity interval training was the chosen one, since it is possible to find studies applying “high intensity interval exercise”; “high intensity intermittent training”; “high intensity intermittent exercise”; “repeated sprint training”; “sprint interval exercise”; among other possibilities.*

**I have incorporated Table 3, as well as the two crucial components of the intervention, namely heart rate and interval time, as per your guidance. These two variables play a pivotal role in defining the exercise intensity and can serve as indicators of whether the experiment aligns with the criteria for HIIT.**

**In the introduction, I have also emphasized the rationale for adopting the term 'HIIT,' underlining why it is the recommended descriptor. The following is my definition and explanation of this issue.**

**High-Intensity Interval Training (HIIT), "high intensity interval exercise," "high intensity intermittent training," and "high intensity intermittent exercise" are terms that are often used interchangeably. They all describe the same fundamental concept: alternating high-intensity exercise with rest intervals or lower-intensity exercise.**

**On the other hand, Repeated Sprint Training is a specific type of training primarily used in sports involving sprinting or short bursts of intensity. It typically involves repeated, brief, maximal-effort sprints with short recovery periods between each sprint. While it shares similarities with HIIT, Repeated Sprint Training is often tailored for athletic events and is particularly focused on enhancing sprinting capabilities.**

**Similarly, Sprint Interval Exercise involves short bursts of maximal effort sprints followed by rest or low-intensity intervals. Like Repeated Sprint Training, it is commonly employed in sport conditioning programs.**

**The term HIIT has achieved widespread recognition as the preferred and widely recognized phrase for this specific training method. This is due to its clarity and consistency. When individuals hear "HIIT," they immediately grasp the training regimen characterized by short, intense bursts of exercise alternated with periods of rest or low-intensity intervals. Over time, it has garnered immense popularity, making it a powerful and easily identifiable concept within the fitness industry.**

**Secondly, HIIT offers simplicity. It is a succinct and clear acronym that effectively summarizes the core elements of this training method. Its ease of recall and application make it highly accessible.**

**Furthermore, HIIT has garnered substantial recognition within academic and research disciplines over the long term. It has become a cornerstone of scientific investigations and studies, solidifying its position as the preferred term for describing this form of training. Researchers and academics frequently rely on standardized terminology to ensure precision and clarity in their work.**

**In summary, these terms all describe training methods that involve alternating periods of high-intensity exercise with rest or low-intensity intervals. The choice of terminology may vary depending on the context, the specific goals of the training program, or the sport or fitness discipline in question. However, the fundamental principles of HIIT remain consistent across these terms.**

I believe that these revisions have significantly improved the quality and clarity of the manuscript.

If further revisions are necessary or if you have any additional questions or concerns, please do not hesitate to contact me. I am committed to ensuring that the manuscript meets the journal's requirements and maintains its academic rigor.

Thank you once again for your time and expertise in this review process. I look forward to hearing from you and the reviewers regarding the outcome of this submission.

Sincerely,

Yixuan Liu
